# The custodian administered research extract server: “improving the pipeline” in linked data delivery systems

**DOI:** 10.1186/2047-2501-2-6

**Published:** 2014-08-18

**Authors:** Tom Eitelhuber, Geoff Davis

**Affiliations:** Data Linkage Branch, The Western Australian (WA) Department of Health, 189 Royal St East Perth, WA 6004 Perth, Australia

## Abstract

**Background:**

At Western Australia’s Data Linkage Branch (DLB) the extraction of linked data has become increasingly complex over the past decade and classical methods of data delivery are unsuited to the larger extractions which have become the norm. The Custodian Administered Research Extract Server (CARES) is a fast, accurate and predictable approach to linked data extraction.

**Methods:**

The Data Linkage Branch (DLB) creates linkage keys within and between datasets. To comply with the separation principal, these keys are sent to applicable data collection agencies for extraction. Routing requests through multiple channels is inefficient and makes it hard to monitor work and predict delivery times. CARES was developed to address these shortcomings and involved ongoing consultation with the Custodians and staff of collections, plus challenges of hardware, programming, governance and security.

**Results:**

The introduction of CARES has reduced the workload burden of linked data extractions, while improving the efficiency, stability and predictability of turnaround times.

**Conclusions:**

As the scope of a linkage system broadens, challenges in data delivery are inevitable. CARES overcomes multiple obstacles with no sacrifice to the integrity, confidentiality or security of data. CARES is a valuable component of linkage infrastructure that is operable at any scale and adaptable to many data environments.

## Introduction

### Data linkage in Western Australia

Linked health data has been used for analysis in Western Australia since the mid-1980s, initially in the form of standalone systems such as the Maternal and Child Health Linked Database [1] and the Road Injury Database [2]. Research program specific systems such as these were phased out after the introduction of the WA Data Linkage System, managed by the Data Linkage Branch (DLB) in the WA Department of Health [3].

**Figure 1:**
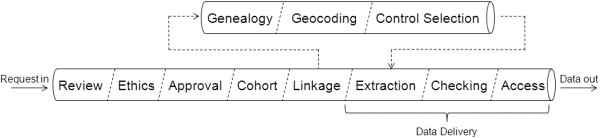
**“The Pipeline”; an illustration of the timeline of a linked data project in Western Australia’s Data Linkage Branch, from initial application; through approvals, cohort creation and linkage; to data delivery.**

The DLB performs routine linkages, value add functions, and facilitates an extract tailoring service to researchers. The DLB performs routine linkages of Department of Health data collections including Hospital Morbidity, Emergency Department, Cancer Registry, Mental Health and Midwives Notifications, as well as non-Health collections such as the Registry of Births, Deaths and Marriages, Road Crash data and the State Electoral Roll. In total, DLB maintains linkage keys for well over 30 different state-based data collections [4], in addition to overseeing value adding processes relating to geocoding and genealogy. The branch also provides a service to researchers by providing tailored extracts of de-identified, linked information about study cohorts, often including multiple cohorts of cases, controls and family connections. Every research request must go through initial review and feedback, ethical approval, custodian and DLB approval, cohort creation, linkage, service data extraction and checking before data is provided. This series of processes form the titular “pipeline”, which is illustrated in Figure 1. The final few phases, wherein the linkable datasets are requested, extracted, standardised, checked and made available to the researcher (for example, through a secure laboratory environment or the provision of aggregate tables or deidentified datasets to the researcher) are collectively referred to in this paper as “data delivery.”

Since DLB’s commencement, projects using linked data have increased significantly in number [5], scope and complexity [6], thereby causing the limitations of pre-existing data delivery methods in Western Australia (herein referred to as the “classical model”) to become increasingly pronounced. The Custodian Administered Research Extract Server (CARES) was created to address these limitations.

The community benefits of data linkage are well established [7]. Consequently data linkage has become an Australia-wide, mainstream resource for population research, with linkage centres in all states as well as a cross-jurisdictional linkage infrastructure. Under these circumstances, the improvements to data delivery systems created by CARES are now relevant at a national level. This paper provides a detailed overview of the DLB’s ongoing consultation with stakeholders, as well as the challenges faced relating to hardware, programming, data handling, governance and security during the development of CARES. It will be of interest to the community directly involved in building and maintaining linkage systems as well as the researchers who access them.

## Background

### Linked data: balancing privacy with utility

Data linkage is the technique of joining together pieces of information thought to belong to the same person, using common demographic fields such as names, dates of birth and addresses [8]. Linked data is relatively inexpensive to assemble compared to traditional survey methods [3]. By connecting information between previously disparate data collections, complex research may be carried out, which would otherwise not be possible. Since its inception, DLB has been mentioned in over one thousand journal articles.

The community benefits of research using linked data include: the ability to conduct longitudinal research studies; epidemiological monitoring and surveillance on a large scale; evaluation of health services; and an alternative to intrusive survey methods of data collection [7].

**Figure 2:**
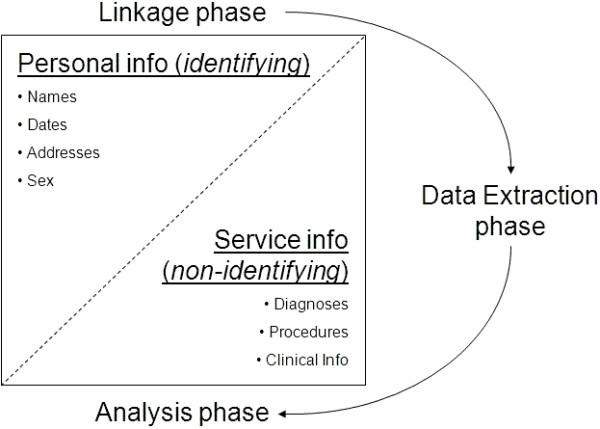
**Visualisation of the separation principle; an illustration of the division of data and related phases of linkage tasks which adhere to the separation principle in the Data Linkage Branch at the Western Australian Department of Health.**

While the benefits of data linkage are by now well illustrated, linking information about individuals from multiple sources raises privacy concerns, [9, 10] so strategies to protect privacy need to be built into all aspects of DLB’s operations. One such strategy is the adherence to the separation principle, a practice in which the demographic and clinical components of a dataset are kept strictly separate. People employed to link the data use the demographic fields to create the links between records, while researchers with ethics approval and custodian permissions use the links and the non-identifying data to perform their analysis [9]. This technique is also used by other Australian linkage centres such as New South Wales’ CHeReL [11] and South Australia/Northern Territory’s SA/NT Datalink [12]. Other models, such as those featuring fully interlinked data repositories or deterministic linkage procedures that avoid the use of demographic data (for example, where a comprehensive “citizen’s ID” is available), have been used by linkage centres in other countries. The separation principle is depicted in Figure 2.

### Data extraction: the classical model

**Figure 3:**
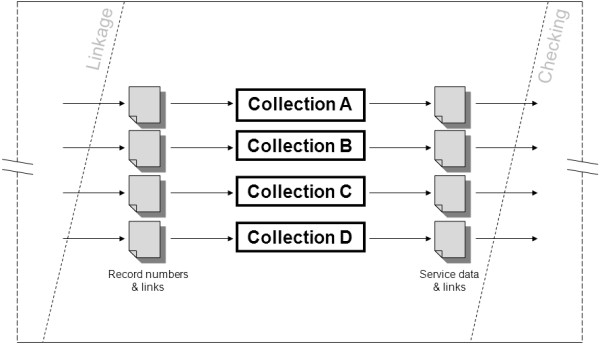
**“Zooming in on the Pipeline”: the Extraction phase under the classical model of linked data extraction; an illustration of the data flow required by the classical (historical) method of linked, de-identified service data extractions, wherein each data collection is responsible for extracting their own data, at the Western Australian Department of Health.**

To adhere to the separation principle, non-identifying data cannot be stored with the identifying data and linkage keys held by DLB. This requirement for separation necessitated a phase of data extraction, as denoted in the diagram, run outside of DLB. In this phase, the created links are appended to non-identifying data extracted by the custodian of each collection relevant to the research request. For example, if a research project requires linked Hospital Morbidity, Emergency Department and Cancer Registry data, the linkage keys for the records in question would need to be sent to these three collections, with accompanying unique identifiers to connect them to the collections’ own data. Figure 3 shows the flow of data through the “Extraction” phase of the pipeline under this traditional methodology.

Note that the files of linkage keys split into a number of separate streams – one per collection – upon provision to the collections for data extraction. This method causes a number of problems:

- ➢ Duplication of efforts – because each collection must carry out their own component of the extraction, the number of staff required will be at least as many as the number of datasets being extracted.
- ➢ Inefficiency – the process can take a long time, due to the number of people involved and range of systems from which data must be drawn, each with independent management structures in place.
- ➢ Predictability – each of these systems is operated in a context of competing priorities, with many being understaffed and facing the pressure of short-notice, short-deadline tasks. In this environment, an extraction of linked data may be deemed a low priority. As such, it is very hard to predict the amount of time it will take to complete an extraction, making it impossible to accurately predict data delivery schedules to those applying for linked data.
- ➢ Instability – an entire project can be slowed to a halt if just one of the participating collections is unable to release data in a timely manner.
- ➢ Post-extraction standardisation – each collection utilises their own software to produce extract files and this software is not necessarily the same from one collection to the next. Even when they are the same, there is no guarantee that the data will be output in an identical way. Therefore, it is necessary for DLB to standardise these extracted files before sending them to the researcher. Time is taken to convert files to a common type, modify fields to match formats (for example, dates may be expressed DD/MM/YYYY in one set and YYYYMMDD in another) and create layout description files for the researcher to use. All of this adds time and work to the process.
- The combined result is a system of data delivery that’s slow, complicated and unreliable, as well as a drain on resources due to the regular input required from staff across multiple departments.

## Methods

### Data extraction: the CARES model

**Figure 4:**
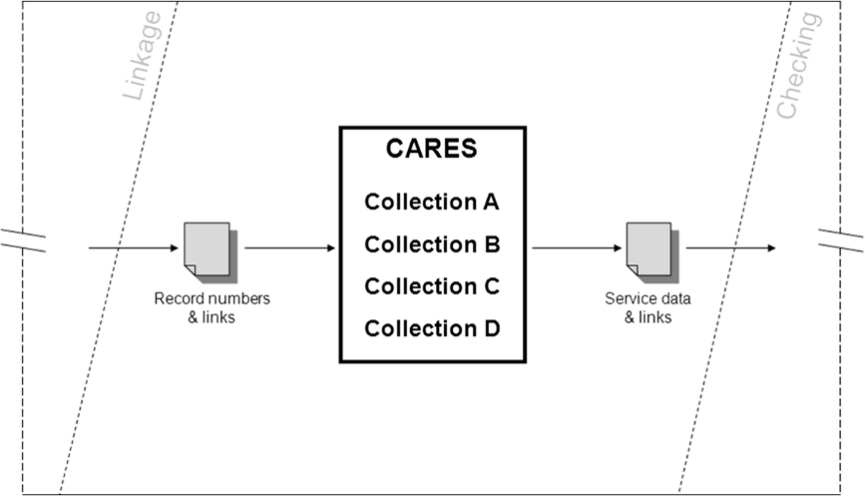
**“Zooming in on the Pipeline”: the Extraction phase under the CARES model of linked data extraction; an illustration of the data flow required by DLB’s proposed method of linked, de-identified service data extractions, wherein partial, regularly updated copies of the data collections are maintained on a central hub.**

The shortcomings of the classical model of data extraction were the motivation to explore alternative approaches to the delivery of linked data. CARES represents a technological response to these shortcomings, replacing the central component of the “Extraction” phase of the pipeline, wherein extract requests are sent to each participating collection, with a unified data hub. This concept is illustrated in Figure 4. CARES would house regularly updated, partial copies of each data collection, such that extraction tasks could be carried out by a single operator using one system.

Due to the collaborative nature of linked data delivery, which sees stakeholders from various sections of the Health Department cooperating and sharing resources, the technological response could not be realised without an accompanying, high-level organisational response. This section of the paper will describe these responses in terms of relationships and collaboration (organisational), systems and processes for data handling (technological), and the CARES model of security and governance (both).

### Strengthening relationships & collaboration: the CARES steering group

While the need for an extract server had been recognised in WA for some time, the project began in earnest during 2011 when initial funding support was received through the multi-agency Developmental Pathways Project [13] funded by the Australian Research Council, led by the Telethon Kids Institute.

A conceptual plan for development of CARES was initially discussed at a meeting with custodians of each of the targeted data collections in 2011. Discussions included security, data transfer and storage, record and person identification and extraction techniques, culminating in the formation of a project steering committee and a detailed knowledge management plan.

Close working environments, long standing collaborations and mutual trust made it possible for custodians as a group to reach an appreciation that CARES could be built and implemented to enhance existing arrangements and provide a powerful new facility over which custodians would retain full control and oversight.

The principle mechanism for governance and management of CARES was established early in the development phase, in the form of a Steering Group, which was created as a decision-making body. The group includes custodians from five of the contributing collections as well as representatives from the DLB. The core duties of the group are to discuss development and implementation, make decisions regarding governance and plan and approve the project’s future activities. The Data Linkage Branch anticipated custodians’ concerns surrounding: security; ethics; operator expertise; translatability of methods; and loss of control over the data and related procedures. This was pre-empted by providing a thorough overview of the CARES model, particularly its adherence to the separation principle, as well as ideas as to how CARES might take shape as a collaborative effort which could benefit the collections as well as the DLB. Six meetings were held over the first 30 months of the CARES project cycle, in parallel with other methods of ongoing liaison, and provided a regular opportunity to report back on positive outcomes and benefits. This open, cooperative approach to project oversight was critical to the enrolment of stakeholders’ enduring support, and consequently brought the project to fruition.

### Strengthening relationships & collaboration: the “Apprenticeship” method

Each data collection is staffed by a team of data analysts. These are the people who run the service data extractions for each collection under the classical model of data extraction. Given that a significant amount of collection-specific knowledge factors into these extractions, it was important to capture and retain the expertise of the analysts in the transition to CARES. In order to preserve knowledge and techniques, the CARES Data Coordinator carried out a series of “apprenticeships” with each of the data collections.

These apprenticeships typically lasted between two and eight weeks, depending on the complexity of the work, and the Data Coordinator’s familiarity with the software being used. The Data Coordinator learnt important background information about each collection, including their history, data sources, loading processes and error checking measures, before moving on to the data extraction methodology. This included how to write extraction scripts, derive custom variables and the important decision making processes for dealing with the criteria in an extraction request. This information was gathered by sitting with analysts and observing their work, before moving on to running data extractions first hand.

The apprenticeships also provided an opportunity to contrast communication with analysts against that with custodians, and made apparent the importance of the former. While custodians hold the power over use of their data and are the key decision makers in areas of governance, their knowledge of the details of day-to-day data operations may be on a different level to that of analysts. Dealing with the analysts directly has been essential due to limitations on Custodians’ availability, but also for the “ground level” perspective they bring, as they are in the best position to explain the minutiae of their day-to-day tasks. This proves valuable with obtaining ongoing detail for the “nuts and bolts” of extraction scripts.

A number of factors influenced the progress of these apprenticeships and the subsequent readiness to channel data through CARES. These included collections’ heavy workloads limiting the amount of time available to work on CARES issues, the level of documentation and organisation surrounding extraction procedures and staff turnover, including at the managerial level. In most cases, factors such as these caused limited delays in progress, however at least one collection was forced to withdraw from CARES indefinitely, emphasising the extent to which CARES’ progress was dependant upon factors beyond the control of DLB and the CARES Steering Group.

As the apprenticeship phase was completed for each collection, the next step was to introduce the data into the CARES infrastructure.

### Data handling: retrieval

Before populating the CARES system with data from the participating collections, decisions were required regarding hardware requirements, data transferral, storage arrangements, record identification, encryption, security and loading methods.

Hardware was selected to maximise storage space and memory to ensure linked data extractions running against very large datasets were as efficient as possible.

Data update schedules to CARES were determined according to the needs of each custodian, in sympathy with other priorities. To facilitate this, a “transfer” partition was created on the CARES server and access was granted to designated staff from each collection. Data updates are quickly and easily transferred through this space.

All loading programs were created and maintained in-house, thereby making it easy to update them to account for changes in the data, such as added or modified fields.

### Data handling: extraction

Data Extraction is *the* critical component of the CARES concept. CARES was proposed to enhance the extraction process, therefore measurable improvements in this area were crucial to justify the infrastructure’s existence.

An intended advantage of CARES was the ability to harmonise the varied software and extraction methods of collections into a single type. This provided a number of challenges. To ensure that the particulars of data extraction were not lost or altered in the transition to CARES, a great deal of consultation and script checking with analysts was undertaken. Under certain project conditions, the collections’ analysts are required to undertake non-standard procedures such as deriving custom fields or filtering selected records. Through ongoing consultation it was ensured that such practices would be possible under CARES, either as inbuilt features or ad-hoc steps that were compatible with the system.

The main goal of CARES has been to expedite the extraction process. This is partly achieved through having all components of an extraction handled by one user. Additionally, much of the extraction process was automated, with each dataset being handled by a dedicated script called a “module”, containing any standard processes.

Each of these modules followed the same basic structure:

1. Prompt the user for their username and password.
2. Import an input file of CARES IDs and linkage keys.
3. Retrieve applicable records from the CARES database.
4. Append the linkage keys to the retrieved standalone dataset.
5. Run any necessary field mapping, filtering, exclusions and creation of derived fields. These optional steps could be triggered by the user.
6. Output the final set of records to a file of standardised format, and create corresponding layout files and quality/validity checking summaries.

### Data handling: quality assurance

Each of the collections has their own quality assurance protocols, run to ensure that data has been extracted correctly and to specifications. The DLB’s Client Services division includes analysts who perform additional checks and standardise the data before it is provided to the researcher.

To integrate these measures into CARES, the custodian and lead analyst from each collection were consulted, as well as the Linkage Branch analysts. Additional checking steps could be requested, and existing ones could be fine-tuned before being hard-coded into the CARES modules. Each module could then produce a checking report for the user, covering correctness of content, date coverage, field groupings, the existence of missing records and more. Additionally, since all datasets coming out of CARES were already in a uniform state, the DLB analyst would not need to standardise any of the files.

### Governance and security

Administrative data collections containing information about individuals are inherently risky. Perhaps one of the more famous examples demonstrating this was the identification of William Weld’s (the governor of Massachusetts) health records occurring in 2002. Not only was the governor’s privacy compromised, but his data may have been sold for profit, as well as handed on to researchers [14]. Some have suggested that this example is not realistic since it was the results of an academic wishing to make a point [15]. However, this view is easily refuted by the significant number of attacks made on data collections of all types involving attempts to obtain money, ‘hacktivism’ attacks, and espionage [16].

The Department of Health has a number of strict policies surrounding the collection [17], accessibility [18] and use [19] of personal health information. There was no question, that with so many sensitive datasets being co-located on CARES, it was vital to put into place multiple levels of governance and security.

The Steering Group directed the provision of data from collections to CARES to be formalised in a Memorandum of Understanding, which outlined the data items and schedule of provision for each dataset, as well as specifying the “ownership” each party had over their data – DLB could not release unit record level data without the permission of the applicable collection(s) and the collections could not release linkage keys without the permission of DLB.

It was also important to maintain strict control over who could access the data on CARES, and under which circumstances [20]. The Steering Group endorsed a three tiered framework to dictate limits on user access:

- ➢ Confidentiality Agreements – each new CARES user is required to sign a standard confidentiality agreement, much like those signed by analysts and linkage officers who access similar data on other systems.
- ➢ Dataset access approvals – each CARES user must have their access to each dataset approved by the applicable Custodian. This is done using a paper form for each user and includes optional access expiry dates.
- ➢ Data use approvals – each task carried out on CARES must have approval given by the custodian of those sets being accessed. This approval can be informal, such as an email of confirmation, and is saved in the applicable task folder. Given that a detailed approval process already exists for official DLB projects, it was relatively easy for CARES approvals to be factored into the existing protocols.

Firm dividing lines were maintained between CARES and other related databases, such as the DLB’s database of linkable demographic data, their created linkage keys and the separate DoH data collections. All of these systems exist completely separately of CARES on different computer servers within the Department of Health. The separation of the data on CARES and the linkage keys was compulsory to maintain adherence to the separation principle. This, combined with CARES’ separation from the demographic and other non-CARES data, ensures that users cannot trivially “draw a straight line” between a record on CARES and a corresponding record in another database (or even another record on CARES which, unknown to the user, belongs to the same person). This eliminates the potential threat of a user discovering additional confidential information about individuals on the system. Without acquiring approval to import project-specific linkage keys into a designated project working space, a CARES user can see no more than isolated, unlinked, deidentified information from only those tables they are permitted to see.

All records on CARES are given a customised “CARES ID” upon loading, generated by encrypting the collections’ own unique IDs. The software on CARES allows for encryption but not decryption, meaning that IDs can be converted at the loading stage but a subsequent user cannot backtrack from the CARES ID to the original one. If a CARES user wishes to contact a collection regarding records in CARES, they can request that one of DLB’s linkage officers decrypt and forward the IDs. This way, there is a bridge between the CARES and the collections’ own systems, however at least one layer of decryption and the involvement of a third party is required to cross it.

Finally, the security of the CARES system was strengthened through restricted physical and electronic access. The partition of the server dedicated to secure file transfer contains subdirectories allocated to each collection or CARES user, with individual access limited to relevant subdirectories. Each file transfer, including data updates and linkage keys for projects, could only be accessed by the file’s sender and intended recipient. Similarly, restrictions are placed on project working directories such that only those users approved by Custodians to work on a given project are granted access to its folder.

### Operation

As the CARES system moved toward live production it was important to maximise confidence in the veracity of outputs. A large number of parallel extraction tasks were run, in which each project saw a CARES version run alongside the true version. The resulting files could be compared to ensure that that both CARES and the Collection had extracted the same records.

These comparisons proved valuable, as in several cases the initial attempt failed. The reasons for discrepancies included differences in exclusion criteria, cut-off dates, missing data and simple script bugs. Over time, these issues were resolved and the CARES and Collections extracts were brought into line. The comparison process also identified errors in the collections own extraction methods and issues relating to data consolidation and the timing of updates. By running these comparisons, problems could be identified and resolved outside of CARES, as well as within.

After passing this rigorous testing phase, the Birth, Death, Electoral Roll, Cancer, Emergency, Hospital Morbidity and Midwives collections were approved for live data extractions from CARES. In the first year of going live, CARES has been used to extract, in part or whole, the data for over 30 projects.

## Results

Improvements in efficiency and predictability of data delivery have been quantified through the tracking of turnaround times.

Four datasets were examined for improvements. Comparisons were made between data extractions which used the CARES model and those which used the classical model, for projects completed between January 2012 and June 2014.

Improvements in efficiency have been measured using mean turnaround time. Turnaround time was defined as the number of elapsed days between an extraction of data being formally requested, and the extracted data file being returned to DLB’s Client Services Division. The figures in Table 1 show that, in the cases of these four collections, running the data extraction using CARES results in a substantial decrease in turnaround time.

**Table 1:** **Mean, first quartile, median and third quartile on turnaround times for data extractions run using CARES and non-CARES methodologies, for projects completed between Jan 2012 and June 2014**

Collection		# projects	Elapsed days from extract request to extract completion			
			Mean	Q1	Median	Q3
A	CARES	39	3.0	1.0	2.0	4.0
	non-CARES	87	31.0	9.0	18.0	34.0
B	CARES	29	3.3	1.0	2.0	4.5
	non-CARES	37	38.4	12.5	32.0	51.5
C	CARES	10	3.9	1.0	2.0	5.0
	non-CARES	21	8.7	1.5	4.0	13.0
D	CARES	13	3.7	1.0	3.0	6.5
	non-CARES	36	13.4	1.0	6.5	17.0

Improvements in predictability were measured using the standard deviation on turnaround time. A lower standard deviation means a smaller “window” of likely completion, such that the completion date can be more accurately predicted. Table 2 shows that, in the cases of these four collections, the use of CARES led to a significant decrease in the standard deviation.

**Table 2:** **Standard deviation on turnaround times for data extractions run using CARES and non-CARES methodologies, for projects completed between Jan 2012 and June 2014**

	Collection	# projects	Std dev
A	CARES	39	3.1
	non-CARES	87	33.4
B	CARES	29	3.4
	non-CARES	37	33.6
C	CARES	10	4.1
	non-CARES	21	12.9
D	CARES	13	3.7
	non-CARES	36	16.8

In July 2014, when CARES had been in use for twelve months, there was opportunity to investigate the impact of CARES across a broader, “whole of project” timeline, which would provide insight into the improvements CARES could make to researcher waiting times. Due to factors that lie outside of CARES’ influence, such as ethics approval and researcher feedback, not all components of the timeline were appropriate for consideration. For this reason, turnaround time was defined as the number of elapsed days between a data extraction’s formal request (the same “start point” as used in Tables 1 and 2) and final dispatch of data to the researcher, therefore including later stages of data delivery such as quality assurance, standardisation of datasets and the creation of supporting documentation.

Table 3 shows these turnaround figures for all DLB projects completed between January 2012 and June 2014, divided into three groups: “None” for projects which did not utilise CARES at all; “Part” for those which used CARES for some, but not all, data extractions and; “All” for those which used CARES for all required data extractions. The table shows that there is a modest improvement in turnaround times for projects which used CARES in part compared to those which did not use CARES at all. However, there is a drastic improvement for those which used CARES in their entirety.

**Table 3:** **Mean, first quartile, median and third quartile on turnaround times (from data extract request to total project completion) for projects run using CARES in full, in part and not at all, completed between Jan 2012 and June 2014**

Proportion of project completed using CARES	# projects	Elapsed days from extract request to project completion			
		Mean	Q1	Median	Q3
None	51	69.7	35	62	97
Part	23	61.3	25	63	77
All	16	39.1	15.5	28	58.5

By centralising the extraction process, each project now only requires one staff member for extraction. The 90 projects comprised by Table 3 included a median of four data collections and a third quartile of six. Extractions run using the classical method would generally require a minimum of one staff member per collection.

By producing uniform formats and file types, CARES has also removed the need for most post-extraction standardisation. By comparison, a project involving each of the participating collections, carried out using the classical method, would require extensive conversion due to the presence of three different file types, likely with differing field formats contained within.

## Discussion

The implementation of CARES involved ongoing consultation with data custodians and analysts, construction of infrastructure, migration of key procedures, the design of an overarching system of governance and security and extensive testing. This process brought to light a number of strengths, as highlighted in the Results section above, but also some limitations of the CARES model remaining to be addressed through further development.

The reduction in the number of staff required to run a data extraction reduces duplication of efforts and is a major strength. The people who run the extractions are required to closely interpret project criteria and write accompanying extraction scripts, and incoming staff have to learn these processes. Relieving the data collections’ staff from having to run extractions for DLB projects is of great benefit as they are already under considerable pressure to address competing demands on their time.

To evaluate the improved efficiency of CARES, it was necessary to compare the delivery times under CARES to those of the classical model. Data on the latter was available from DLB’s records back to 2008, however it was ultimately decided to focus on those from 2012 onwards. This was done for two reasons: first, to increase likelihood that the time-specific circumstances surrounding the extractions would be similar for CARES and non-CARES projects, thereby avoiding possible confounding of results; second, to include a similar number of “non-CARES” and “part/all-CARES” projects in the results. When examining the 2012-onwards and 2008-onwards stats side by side, one of the data collections showed a larger average turnaround time and larger standard deviation for the latter (possibly due to some time-specific circumstances prior to 2012). However, the remaining results were largely similar.

If the efficiency improvement shown in Table 1 is maintained, the DLB will be in a position to deliver data to researchers in a more timely fashion, removing the current delay issues to research using linked data from the collections stored within CARES. If the predictability improvements shown in Table 2 are maintained, the DLB will be in a position to more accurately estimate delivery times to researchers for these projects. The time difference shown in Table 3 between projects which used CARES partially versus totally suggests that, should CARES continue to grow and incorporate more data collections, significant improvements in efficiency and stability can be expected. This should be considered especially important in the light of a current limitation of CARES revealed by these results – projects which include any data collections outside of CARES can still fall victim to long wait times if those external collections take a long time to complete their part of the process. While substantial improvements were seen for projects wholly extracted via CARES, the result was less impressive where projects included collections not yet working with CARES.

It is important to note that CARES only directly impacts certain parts of the pipeline of a linked data project. Factors over which CARES bears no influence, such as complex linkage requirements and holdups in application and ethics approvals, have the potential to blow out project timelines regardless of the presence of CARES. However, there are phases of the pipeline outside of data extraction which CARES has been able to improve. Due to the simplification of post-extraction requirements projects have begun clearing the final phase of checking and standardisation more rapidly before data is released to the researcher. There have also been reports from the DLB’s Data Linkage team that, for projects making use of CARES, sending their files of linkage keys to a single destination makes for a simpler, more streamlined process. Finally, there is potential for CARES to be used to select study cohorts and respond to queries in the earlier phases of the pipeline. This could create similar benefits of efficiency, predictability and workload reduction to those seen in the extraction phase. Although numbering too few for proper analysis, early uses in this respect have shown promise.

The path to making the CARES system a reality could easily have been one of numerous setbacks and delays. Decisions were made during the project lifecycle to maximise the likelihood that key stakeholders bought into the concept and facilitated rapid progress throughout. On the surface, some could argue that the best model of data delivery would involve a fully linked data repository, wherein all linkage, extraction and even data analysis could take place in a single data warehouse containing joined up, multi-agency information – both personal and clinical – about the full state population. While such an integrated system would remove practically all inefficiencies of the classical model of data delivery, it would have encountered difficulty sitting within the policy and political framework affecting data linkage within Western Australia, and indeed much of Australia. Consider, for example, its incompatibility with the privacy and confidentiality guidelines in the Cross Portfolio Statistical Integration Committee’s *High Level Principles for Data Integration Involving Commonwealth Data for Statistical and Research Purposes*[21] and the requirements regarding separation of identifiers from content in the Australian Bureau of Statistics’ *Challenges of Statistical Data Integration*[22]. As such, it was not considered a suitable option for the DLB. Additionally, CARES’ adherence to the separation principle was an important factor for the arrangement to be palatable to data custodians. By demonstrating multiple measures to mitigate risk, and surrounding these measures in a development process that valued communication, collaboration and robust quality checking, it became trivial for custodians to be able to place their trust in CARES. It is highly unlikely that a fully integrated data warehouse would ever have received the same level of support.

The DLB’s long history is significant to the challenges experienced (and avoided) during the development of CARES. The linkage and extraction infrastructure in Western Australia has evolved from systems originally developed in the nineties and CARES has been created within an environment where these systems are well established, proven and accepted. The DLB has collaborated and been physically co-located with the Department of Health data collections for many years, creating trust and rapport among their combined staff. While challenges in the efficiency, predictability, stability and burden of linked data delivery are likely to be encountered by other agencies, in Australia and internationally, each will have their own relationships, infrastructure, policy situation and historical context to consider when creating solutions. CARES demonstrates a privacy preserving approach to these issues which may be of value to other linkage infrastructures, particularly those which follow a similar blueprint to the DLB and those operating under similar circumstances.

## Conclusions

The WA Department of Health’s Strategic Intent includes the following aim: “Remaining at the forefront of international medical research by investing in infrastructure and building partnerships with industry and individual researchers [23].” CARES is contributing to this goal by improving the efficiency of data delivery systems, and has been partially supported by key industry partners. By doing so it allows DLB to better facilitate the important research carried out using linked data. Enabling and promoting the use of linked data for research and evaluation of services is the responsibility of linkage centres throughout Australia, and indeed is a tool of significance on an international scale. CARES demonstrates a technological approach which is achievable within existing policy frameworks. While the exact data flows and systems employed will be specific to individual linkage centres, the overarching strategy, to centralise extraction processes whilst avoiding a large interlinked repository, is reproducible anywhere. The other major contribution of CARES, to a wider audience (and arguably a bigger challenge than the purely technological endeavour) is the multi layered strategy for achieving stakeholder enrollment in the idea and now, years later, continued custodian enthusiasm and active support of the infrastructure. The CARES approach is not the only means of achieving efficiencies of centralisation and will not be the only approach taken. However, alternatives such as large interlinked repositories are simply not acceptable in many existing ethical and policy frameworks. For those unable to contemplate such an approach, CARES offers the global linkage community an additional strategy to realise increased benefits from linked data research.
